# Gut Microbiota and Microbial Metabolites in the Regulation of Chemotherapeutic Resistance

**DOI:** 10.3390/cells15211975

**Published:** 2026-10-29

**Authors:** Shontol Bhowmic, Pritha Roy Choudhury, Prodipto Das, Saptak Banerjee, Jayanta Chakrabarti, Subir Kumar Juin, Sweta Ghosh

**Affiliations:** 1 Department of Surgical Oncology, Chittaranjan National Cancer Institute (CNCI), Kolkata 700026, India; 2 Department of Immunoregulation and Immunodiagnostics, Chittaranjan National Cancer Institute (CNCI), Kolkata 700026, India; prithadata27@gmail.com (P.R.C.); prodiptodascnci@gmail.com (P.D.); drsaptakbanerjee@gmail.com (S.B.); 3 Department of Zoology, University of Calcutta, 35 Ballygunge Circular Road, Kolkata 700019, India; subirjuin.vbu@gmail.com

**Keywords:** chemoresistance, gut microbiota, microbial metabolite, dysbiosis, chemotherapy

## Abstract

Resistance to chemotherapy continues to be a significant challenge for cancer treatment and a contributing factor to treatment failures and tumor progression. Recently, the gut microbiome has emerged as an important modulator of the response to chemotherapy. Numerous pre-clinical and clinical studies have demonstrated a direct association between gut microbiota composition and therapy effectiveness in many cancer types, such as colorectal, breast, pancreatic, gastric, and lung cancers, melanoma, and hematological cancers. Gut microbiota effectively regulate the efficiency of chemotherapeutic agents in various ways, either by biotransformation of chemotherapeutic agents or modulation of host immunity. In addition, microbial metabolites such as short-chain fatty acids, secondary bile acids, and tryptophan metabolites are also involved in the regulation of chemotherapy response through modulating apoptosis, immunity, and epithelial–mesenchymal transition. Finally, dysbiosis induced by chemotherapy and antibiotics has also been reported to reduce the effectiveness of therapy. This review will emphasize the ways in which gut microbiota can modulate chemotherapeutic response and induce chemoresistance. Understanding the interactions between chemotherapeutic drugs and the gut microbiota can help to enhance the efficiency of chemotherapy and find more appropriate personalized treatments.

## 1. Introduction

Cancer is a major global health disease, and chemotherapy remains one of the essential therapeutic strategies against cancer. Chemotherapeutic drugs are primarily cytotoxic, and they are designed to inhibit cancer cell growth by destroying rapidly dividing cells. Chemotherapeutic drugs can be of several types depending on their mode of action, such as antimetabolites, alkylating agents, mitotic inhibitors, topoisomerase inhibitors, and others. Several chemotherapeutic drugs, such as 5-fluorouracil (5-FU), oxaliplatin, vincristine, cisplatin, doxorubicin, and irinotecan, are being widely used to treat several types of cancers [1,2]. However, there are several challenges associated with chemotherapy, including the side effects of the drug. The most common side effects include fatigue, diarrhea, nausea, loss of appetite, mucositis, vomiting, and hair loss. Though significant advancements have been achieved through chemotherapy, resistance to chemotherapeutic drugs is still a huge challenge in the field of cancer research. It is also the sole reason for most of the relapse cases in cancer patients. Chemotherapeutic resistance can be either intrinsic or extrinsic. Intrinsic chemoresistance is pre-existing and is exhibited by neoplasms before exposure to chemotherapeutic drugs. Several factors are responsible for intrinsic chemoresistance, such as mutations in genes regulating the KEAP1-NFE2L2 pathway; breast cancer stem cell-driven (BCSCs) chemoresistance; activation of intrinsic pathways such as MAPK, PI3K/Akt, and hedgehog signaling, etc., and inadequate intracellular drug concentration [3,4]. Acquired chemoresistance arises after the patient is exposed to chemotherapeutic agents, through genetic and epigenetic mechanisms. Common epigenetic mechanisms through which acquired chemoresistance takes place include DNA methylation, histone modification, long non-coding RNA (lncRNA), and miRNA-mediated regulation. Intrinsic and acquired resistance often happen together. Mechanisms by which chemoresistance occurs include: tumor microenvironment (TME) mediated chemoresistance; ATP-dependent drug efflux; drug activation and inactivation; alterations in drug targets; enhanced DNA repair and impaired apoptosis; and failure of cell cycle arrest regulation. Currently, several studies have reported a close relationship between the gut microbiota, microbial metabolites, and chemotherapeutic resistance. The human gastrointestinal (GI) tract is the second-largest surface area of the human body, and it acts as an interface between the body and the external environment. The GI tract is continuously exposed to food, microbes, and antigens. Inside the gut, there is a community of bacteria, viruses, archaea, and unicellular eukaryotes. Genomic data from different studies identified 2172 microbial species in human beings [5]. They were classified into 12 different bacterial phyla and one archaeal taxon, of which 93.5% of species belonged to Proteobacteria, Firmicutes, Actinobacteria, and Bacteroidetes. Of all the organisms listed above, 386 organisms are strict anaerobes that live in the mucous membranes, mainly the mouth cavity and the GI tract. In maintaining the health of the gut, the body relies on the gut epithelial barrier, the immune system, and the gut microbiota [6,7]. The gut microbiota facilitates the development of GALT, IgA-secreting B cells, and T cell populations including Th17 cells and regulatory T cells. Gut microbiota metabolize complex carbohydrates from host food sources to produce several metabolites, including short-chain fatty acids (SCFAs) such as butyrate, acetate, and propionate, and synthesize vitamins like B12, folate, K, riboflavin, biotin, thiamine, and pyridoxine. Microbiota compete with pathogens for nutrients and binding sites. However, these protective mechanisms can be disrupted by altered microbial composition, known as ‘dysbiosis’. The intestinal microbiota has a great influence on peripheral organs in the body, and thus its composition influences various diseases. The gut microbiome also has the proven capability to metabolize xenobiotics and drugs. A recent study has demonstrated that a gut microbial metabolite, p-cresol, can reduce the capacity of the liver to metabolize acetaminophen due to competitive inhibition of hepatic sulfotransferases [8]. Several cancers have been shown to be related to gut microbial dysbiosis in global cancer research studies. Gut bacterial strains, including enterotoxigenic *Bacteroides fragilis* and *Escherichia coli*, are known to cause chronic inflammation and tumorigenesis in colorectal cancer (CRC) patients [9]. Intestinal bowel disease (IBD) is also one of the most well-established risk factors for progression of CRC. Gut dysbiosis is a common event for both IBD and CRC. Like IBD, colorectal tumors display an impaired intestinal gut barrier, which might be the suspected reason for intestinal colonization by oral microbiota. *Helicobacter pylori*, a flagellated Gram-negative bacterium, is responsible for oncogenesis in gastric adenocarcinoma and also alters the proportion of other beneficial bacteria in the GI tract. Lung cancer patients show altered microbial diversity with increased abundance of *Streptococcus*, *Veillonella*, *Thermus*, and *Legionella* [10]. Higher microbial diversity has been associated with better treatment responses. In cervical cancer, HPV infection together with microbes like *Prevotella*, *Porphyromonas*, and *Enterococcus* contributes to carcinogenesis by creating an inflammatory microenvironment. Breast cancer tissues commonly contain HPV and bacterial groups including *Proteobacteria*, *Firmicutes*, *Actinobacteria*, and *Haemophilus influenzae*, which may influence tumor progression and immune responses.

This review examines how gut and tumor-associated microorganisms and their metabolites modify cytotoxic chemotherapy response. We focus on mechanistic evidence for drug biotransformation, immune regulation, biofilm formation, cancer stem-cell programs, epithelial–mesenchymal transition, and transporter regulation; compare evidence across cancer types; and assess the opportunities and limitations of microbiome-directed interventions. Evidence concerning immune checkpoint inhibitors is included only where it clarifies shared microbiome-immune mechanisms and is distinguished from cytotoxic chemoresistance.

## 2. Gut Microbiota: Composition and Functions

The human gut microbiota is an aggregate of a large number of microbes that include bacteria, fungi, archaea, and unicellular eukaryotes. The gut microbiota is concentrated in the distal intestine and acts as an “anaerobic bioreactor” where plant-derived indigestible polysaccharides are metabolized. Apart from their metabolic role, these microbial communities also have structural and protective functions inside the GI tract. Broadly, gut dysbiosis refers to an imbalance in microbial community structure or function. Increasing the abundance of beneficial microbes while reducing harmful ones may help restore microbial balance and promote greater microbial diversity, with higher alpha diversity generally associated with eubiosis and lower alpha diversity often linked to dysbiosis. Firmicutes (Bacillota), Bacteroidetes (Bacteroidota), Actinobacteria (Actinomycetota), and Proteobacteria (Pseudomonadota) are among the four primary classes of bacterial phyla represented in the human gut [11]. In the case of CRC, *Fusobacterium nucleatum*, enterotoxigenic *Bacteroides fragilis,* pks-positive *Escherichia coli*, *Streptococcus gallolyticus*, *Enterococcus faecalis*, *Parvimonas micra*, and *Prevotella intermedia* may be enriched or redistributed. Several of these organisms can also occur at low abundance in healthy individuals; therefore, their disease relevance depends on relative abundance, strain-level virulence, spatial localization, and host context [6,12]. A study conducted by Suzuki et al. discovered that the *Firmicutes* population in the human gut increases with latitude, whereas the *Bacteroides* population in the human gut decreases with latitude. The findings were consistent with sex, age groups, and detection methods [13].

Gut microbiota play some crucial metabolic roles in the human GI tract. They possess certain enzymes that help in metabolizing indigestible food components because humans lack those enzymes. These microbes have co-evolved with humans. The activities of gut microbes can be classified into four categories that interact with each other. The first category is metabolic functions such as fermentation of complex carbohydrates to SCFAs, bile acid metabolism, tryptophan metabolites, polyamines, vitamins, and trimethylamine synthesis. The second category is xenobiotic function, which originates from the activity of microbes that activate, deactivate, or alter the disposition of the drug. The third category is immune function, such as IgA induction and Th17 cell development. Finally, the barrier functions include mucin induction, antimicrobial peptides, and tight junction programming. Xenobiotic function originates from the activity of microbes that activate, deactivate, or alter the disposition of the drug. Classifying these domains prevents one from making assumptions that any taxonomic change will result in one particular functional consequence.

In our ancestors’ time, a large portion of the diet included high dietary fiber and complex carbohydrates. This explains why there is a decrease in microbial diversity in the human gut in recent times. Some of the major substrates in the diet targeted by gut microbiota are complex carbohydrates, polyphenols, bile acids, choline, indole, and amino acids. Fermentation of complex carbohydrates in the intestine by the microbiota leads to the formation of short-chain fatty acids (SCFAs). Butyrate, acetate, and propionate form the major portion of SCFAs. Interaction between SCFAs and G-protein-coupled receptors Gpr41/43 located in gut enteroendocrine cells leads to PYY secretion and promotes nutrient absorption [14].

Apart from carbohydrate fermentation, gut microbiota is also responsible for the metabolism of tryptophan and bile acids. The microbial metabolism of tryptophan leads to the formation of several indole compounds (indoles, kynurenine) that can function as AhR ligands and modulate immunity and inflammation in the intestine. Neuroendocrine functions of the gut are regulated by microbiota through balancing of serum serotonin and catecholamines. Bile salt hydrolases (BSHs) and 7α-dehydroxylases from the gut microbiota convert primary bile acids to secondary bile acids. The secondary bile acids regulate lipid metabolism through the FXR signaling pathway and reduce pathogen colonization, such as *Clostridium difficile*. Gut microbes also metabolize dietary choline through enzymes such as TMA-lyase, resulting in the formation of trimethylamine (TMA) [15]. TMA is subsequently converted into trimethylamine N-oxide (TMAO) in the liver. Alterations in this metabolic pathway have been associated with cardiovascular diseases, colorectal cancer, and metabolic dysfunction-associated steatotic liver disease.

Another important function of the gut microbiota is its involvement in xenobiotic and drug metabolism. Gut microbial metabolites influence drug efficacy and toxicity of drugs by modifying drug metabolism and availability. For example, p-cresol competes with sulfotransferases and inhibits the metabolism of acetaminophen. *Eggerthella lenta* inactivates digoxin by upregulating a two-gene (cgr) cytochrome-encoding operon [16]. Gut microbiota aids in inhibiting pathogen colonization in the GI tract as well. Abundance of commensal bacteria can restrict the colonization of invasive pathogens; e.g., in the case of recurrent *Clostridium difficile* infection (rCDI), it has been observed that microbial diversity is reduced markedly. Microbial influence can also reduce *Clostridium difficile*-induced colitis [17]. Increased association between the human host and microbes stimulates Paneth cells in the gut epithelium to secrete antimicrobial proteins, i.e., RegIIIγ or HIP/PAP, that protect the gut lining from the penetration of invasive microbes.

Besides its metabolic functions, the gut microbiota is involved in the regulation of both innate and adaptive immune responses and plays a major role in maintaining intestinal epithelial barrier integrity. Beneficial intestinal microbes orchestrate immunomodulation through both innate and adaptive immune arms. Butyrate, a microbe-derived short-chain fatty acid, increases Foxp3 expression via its HDAC inhibition activity. Foxp3 determines the population of regulatory T-cells (T_reg_), thus maintaining immune homeostasis. Gut microbiota promotes secretory IgA (sIgA) production that coats certain pathogenic microbes and prevents them from colonizing inside the gut, while facilitating the colonization of beneficial bacteria. Furthermore, one of the major functions of the gut microbiome is to preserve the gut barrier integrity. The gut microbiota stimulates goblet cells to produce mucus in the GI tract [6,7]. This explains why the large intestine has a thick layer of mucus as compared to the small intestine, because in the small intestine, the number of microbes is less. Through the TLR-2 signaling pathway, gut microbiota helps to maintain the gut epithelial barrier via desmosome formation and enhancement of tight junctions.

Several factors affect gut microbiome composition throughout life. Starting from the delivery of a neonate up to adulthood, they are exposed to various microbes that constitute their gut microbial community. Mode of birth significantly shapes the establishment of the infant gut microbiota. Infants that undergo a normal delivery process acquire maternal microbes, including *Lactobacillus* and *Bacteroides*. This creates a diverse microbial community in their gut. In contrast, the GI tracts of C-section infants are colonized primarily by environmental microbes, resulting in reduced levels of beneficial anaerobes, delayed microbial development, and increased abundance of *Clostridium difficile*. Diet is the key determinant of gut microbial composition. Plant-based diets increase microbial diversity and the abundance of carbohydrate-fermenting bacteria such as *Ruminococcus*, *Roseburia*, and *Eubacterium*, while Western diets are associated with increased *Bacteroides*, reduced beneficial microbes, and greater protein fermentation, which can generate harmful metabolites [18]. Dietary changes can rapidly alter gut microbial composition and function, which highlights the strong influence of nutrition on gut health. Antibiotics remarkably alter gut microbiota as they eliminate both harmful and beneficial microbes in a non-specific manner. Reduced microbial diversity may impair bile acid and serotonin metabolism, which increases susceptibility to pathogens such as *Clostridium difficile*. Several antibiotics, including clindamycin, clarithromycin, metronidazole, ciprofloxacin, and vancomycin, can cause long-term changes in microbial composition, reducing beneficial bacterial taxa such as *Bacteroides*, *Actinobacteria*, *Ruminococcus*, and *Faecalibacterium*, while favoring potentially harmful ones [19]. In addition, widespread usage of antibiotics in livestock farming has raised concerns about their harmful effects on humans via food exposure.

## 3. Interaction Between Gut Microbiota and Chemotherapeutic Drugs

Gut microbiota, a miniature community in the human colon encompassing about 3.8 × 10^13^ bacteria encoding hundreds of times more genetic information than the human genome, is reported as an active modulator in chemotherapy rather than a passive participant. Across pan-cancer scenarios, gut and tumor-associated microbial communities modulate nearly every major class of chemotherapeutic drugs, i.e., antimetabolites (gemcitabine, 5-fluorouracil), platinum-based neoplastic agents (cisplatin, oxaliplatin), alkylating agents (cyclophosphamide), etc., by altering systemic drug exposure, efficacy, and toxicity profiles [20,21]. The gut microbiota exerts its modulation in chemotherapeutic intervention in multidimensional facets. Several studies have indicated two-way aspects between microbiota and chemotherapeutic intrusion. Alexander et al. have introduced the “TIMER” hypothesis that extensively demonstrated the host and tumor-associated microbiome interaction based on translocation, immunomodulation, metabolism, enzymatic digestion, and reduced pathobiont diversity and ecological variation [22]. A study in a mouse model showed that cyclophosphamide- a well-known chemotherapeutic drug with an immunomodulatory role- was solely dependent on the translocation of certain gut microbiota to systemic organs such as the spleen, lymph nodes, etc. The drug ruptured the barrier protecting the small intestine. This event influenced the entry of several gut and tumor-associated microbial communities like *Lactobacillus* sp., *Enterococcus hirae*, etc. into the secondary lymphoid organs via the rupture and, in turn, elevated the memory T-helper 1 and T-helper 17 response. Ultimately, it resulted in upregulation of antitumor activity in cyclophosphamide-resistant tumors [23]. A similar interaction was observed in specific commensal microbiota, *Ruminococcus* sp. and *Alistipes* sp., that induced TLR4-mediated priming of innate myeloid inflammatory responses, resulting in improved CpG oligodeoxynucleotide treatment via the TLR9 signaling pathway in lymphoma and myeloma mouse models [24,25]. Studies also deciphered similar bidirectional interactions via gut microbiome-secreted metabolic repertoire modulating the chemotherapeutic efficacy. A commensal bacterium, *Lactobacillus acidophilus,* induces myeloid cells by upregulating NADPH oxidase 2 and reactive oxygen species generation in macrophages, which elevates oxaliplatin-mediated DNA damage via influencing epigenetic alterations. In vitro experiments have indicated that butyrate may improve the response to 5-FU, gemcitabine, irinotecan, and cisplatin treatment via epigenetic and apoptotic mechanisms, although dosage, tumor genetics, time of exposure, and the lack of immune system involvement affect the clinical interpretation.

In addition to their effects on the host, gut and tumor bacteria can modify the structure of anticancer agents through reactions that are independent of host metabolic processes. However, crucially, the nature of the effect is not necessarily inherent to a bacterial species, but depends on the substrate and the enzyme involved. For instance, the bacterium E. coli can activate the prodrug -(aziridin-1-yl)-2,4-dinitrobenzamide (CB1954) via the action of bacterial nitroreductase, whereas bacterial enzymes can also reduce the concentration of gemcitabine [26]. Instead of a general action of E. coli bacteria, gammaproteobacterial long-form cytidine deaminase reduces gemcitabine to inactive 2′,2′-difluorodeoxyuridine [27]. Also, the bacterium *Raoultella planticola* can deglycosylate doxorubicin in the nematode *C. elegans* [28]. Hence, such examples should be interpreted in terms of specific strains, enzymes, drugs, and models used.

However, much evidence regarding biotransformation of chemotherapeutic drugs suggests the negative role of the gut microbiome in therapeutic efficacy (Table 1). One widely held example is gemcitabine resistance in the presence of a Gammaproteobacteria population in pancreatic adenocarcinoma patients. Geller et al. explored the interaction between intratumoral bacteria and gemcitabine resistance. They delineated the role of Pseudomonadaceae and Enterobacteriaceae in deamination of the drug into its inactive form, 2′,2′-difluorodeoxyuridine (dFdU), via cytidine deaminase-mediated metabolism [29]. More recent structural work also established direct conversion of gemcitabine to dFdU by crystallographic study of cytidine deaminase from *Enterococcus faecium* in gallbladder malignancy. The drug resistance and enzymatic activity were eliminated by altering catalytic residues of the associated bacterial enzyme [30]. These findings establish biochemical plausibility but do not yet define the prevalence or effect size of this mechanism in unselected patients.

The development of selective bacterial GUS inhibitors (such as uronic isofagomine derivatives and TCH-3562) that are 4–5 orders of magnitude more selective for bacterial than human GUS and that lessen diarrhea in animal models without sacrificing systemic SN-38 exposure or antitumor efficacy has been spurred by this mechanism [31]. This mechanism is therefore classified primarily as microbiota-mediated toxicity rather than chemoresistance.

Aside from direct biotransformation, the gut microbiome also exerts various pharmacokinetic mechanisms and modulates systemic drug distribution and exposure. They modulate the core drug-metabolizing enzymes, i.e., cytochrome P450, UDP- glucuronosyltransferases, etc., and also drug transporters like P-glycoproteins [38]. The enterohepatic system delivers gut microbiota-produced metabolites (short-chain fatty acids, polysaccharide A, etc.) to the vicinity of hepatic immune cells. Once stimulated by these metabolites, the immune cells, particularly Kupffer cells, produce pro-inflammatory cytokines like TNFα, IL-12, IL-6, etc. In addition, the cytokines stimulate the activity of the NF-κB pathway, which leads to the expression of P450 genes. According to Alam et al., individual differences in the gut microbiome play a significant role in tamoxifen pharmacokinetics, signifying the fact that variability in pharmacokinetics due to gut microbiota is not limited to infused cytotoxic drugs only but also to oral cancer treatments [39].

Enterohepatic recirculation represents an additional mechanism of microbial influence on drug disposition. Bacterial β-glucuronidase, beyond its established role in SN-38 reactivation, deconjugates glucuronidated metabolites of numerous other drugs, thereby prolonging systemic half-life and altering the equilibrium between hepatic elimination and intestinal reabsorption. Given the substantial inter-patient variability in β-glucuronidase and other microbial enzymatic activity, fecal microbial enzyme activity has been proposed as a more mechanistically informative biomarker of chemotherapy pharmacokinetics and toxicity risk than 16S taxonomic profiling alone [31,32].

Cytotoxic chemotherapy transforms the host microbiome, destroys gut microbial ecology, alters microbial metabolic functions, and triggers profound intestinal dysbiosis, which ultimately acts as a proximate driver of gastrointestinal toxicity during treatment among various types of cancer. An overall reduction in the number and diversity of bacteria was reported with the uptake of 5-FU in preclinical models [40]. Along with that decrease in the Bacteroidetes/Firmicutes ratio, reduced Tenericutes, Proteobacteria, and Cyanobacteria were also observed, with increasing *Actinobacteria* and *Verrucomicrobia* leading to 5-FU-induced intestinal mucositis (weight loss, bloody stool, diarrhea, colonic shortening, etc.). In a study, transplantation of fecal microbiota from healthy donors to mice improved different phenotypes caused by mucositis, whereas transplantation of microbiota exposed to 5-FU produced toxicity in naïve recipients and developed dysbiosis in chemotherapy-induced gut injury [41]. Simultaneously, patients with doxorubicin- and 5-FU- based chemotherapy reported that a reduction in *Streptococcus*, *Gemella*, *Actinomyces*, *Veillonella*, and *Granulicatella* (Health-associated commensals) was observed with severity of oral mucositis, but there was an increase in Gram-negative bacteria like *Fusobacterium nucleatum* and *Prevotella oris*. This phenomenon resembles inflammation-driven dysbiosis without administration of antibacterial drugs. Similar shifting within the Proteobacteria phylum was reported in chemotherapy regimens containing 5-FU, folinic acid, capecitabine, irinotecan, and oxaliplatin in colorectal cancer patients [42]. Molecular investigations revealed a correlation between Toll-like receptor (TLR) signaling and chemotherapy-induced dysbiosis. A decrease in Gram-positive bacteria with an increase in Gram-negative bacteria populations raises luminal lipopolysaccharide, triggering TLR-2/TLR-4-MyD88/TRIF-TRAF6 pathways and NF-κB-driven inflammation, leading to mucosal barrier injury.

## 4. Role of Gut Microbes in Chemotherapeutic Resistance

**Figure 1:**
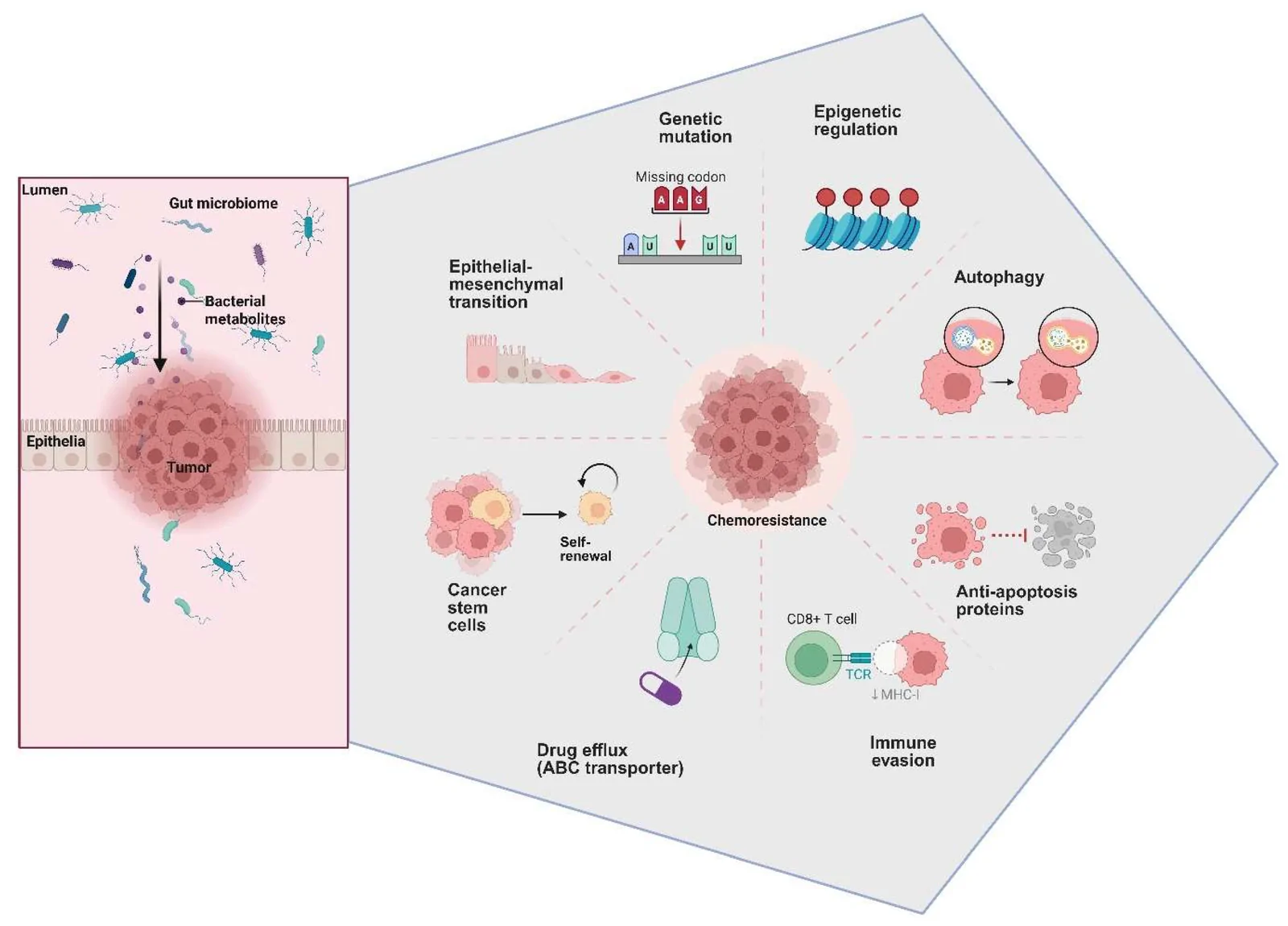
Role of the gut microbiome in cancer chemoresistance. Gut microbiota and their metabolites may influence chemoresistance through multiple mechanisms, including genetic mutations, epigenetic regulation, autophagy, upregulation of anti-apoptotic proteins, immune evasion, increased drug efflux through ABC transporters, cancer stem cell self-renewal, and epithelial–mesenchymal transition (EMT). These mechanisms collectively contribute to tumor survival and reduced sensitivity to chemotherapy.

Gut microbiota and their metabolites can influence cancer chemoresistance through several mechanisms, including genetic and epigenetic alterations, autophagy, immune evasion, drug efflux, and epithelial–mesenchymal transition. These mechanisms, along with increased cancer stem cell self-renewal and anti-apoptotic signaling, contribute to tumor cell survival and reduced chemotherapy sensitivity (Figure 1). Microbiota and tumor-associated microorganisms affect innate and adaptive immune responses via microbial-associated molecular patterns, metabolic compounds, and epithelial signals. Among possible mediators of chemotherapy resistance are dendritic cells, macrophages, neutrophils, natural killer cells, myeloid-derived suppressor cells, CD8^+^ T cells, Th17 cells, and regulatory T cells. According to animal data, *F. nucleatum* induces TLR4/MYD88 pathway activation, autophagy induction, inhibits cytotoxic immune response, and recruits myeloid-derived suppressor cells. On the contrary, specific commensals can help in the development of dendritic cells and effector T cells. Short-chain fatty acids have epigenetic functions, as butyrate, which inhibits histone deacetylase and induces Foxp3^+^ regulatory T cell differentiation. However, their overall impact depends on the concentration, organ environment, tumor genotype, and immune system features [43,44,45,46]. PD-1/PD-L1 axis control is discussed in the context of immune escape. Immune checkpoint blockade response is not considered as evidence of cytotoxic chemotherapy resistance.

Lipopolysaccharide from Gram-negative bacteria can activate TLR4-MYD88/TRIF-mediated signaling followed by downstream activation of NF-κB, STAT3, and MAPK signaling pathways, which in turn increase levels of IL-6, TNF-α, IL-1β, and IL-23. Continuous signaling can cause cell survival, angiogenesis, immunosuppression, and chemoresistance [35,47,48,49]. In CRC models, *Fusobacterium nucleatum inhibits* miR-18a* and miR-4802, promotes autophagy, and causes chemoresistance to 5-FU and oxaliplatin [35]. Microbial biofilms represent an alternative mechanism: the extracellular polymeric substance of biofilms can modify diffusion of drugs, preserve proximity between microbes and the epithelium, and maintain inflammatory signaling [50]. Biofilms formed from different microorganisms, such as *F. nucleatum*, enterotoxigenic *B. fragilis*, and pks-positive *E. coli,* can promote mucosal invasion and either genotoxic or inflammatory damage. This evidence is biologically plausible, although there is no direct evidence that inhibition of the formation of such biofilms reduces chemoresistance in humans.

The impact of microbes on the CSC and EMT programming needs to be distinguished from drug metabolism directly by these microbes. In CRC models, *F. nucleatum* can induce TLR4/MYD88/NF-κB and Wnt/β-catenin signaling pathways, while enterotoxigenic *B. fragilis* stimulates the IL-17/STAT3 pathway, and all three pathways may contribute to stemness-like features. In gastric models, H. pylori can induce Wnt/β-catenin, Hedgehog, Notch, and TGF-β/SMAD signaling pathways, with elevated levels of CD44, LGR5, SNAIL, TWIST, or ZEB1 in different models [29,35,51,52,53,54,55,56,57,58,59,60]. Such markers and pathways cannot be considered absolute proof of CSC status and EMT, and the majority of data is obtained using cell lines and animals. Moreover, secondary bile acids and butyrate might affect the function of stem cells, but it depends on specific conditions.

## 5. Microbial Metabolites Involved in Chemoresistance

**Figure 2:**
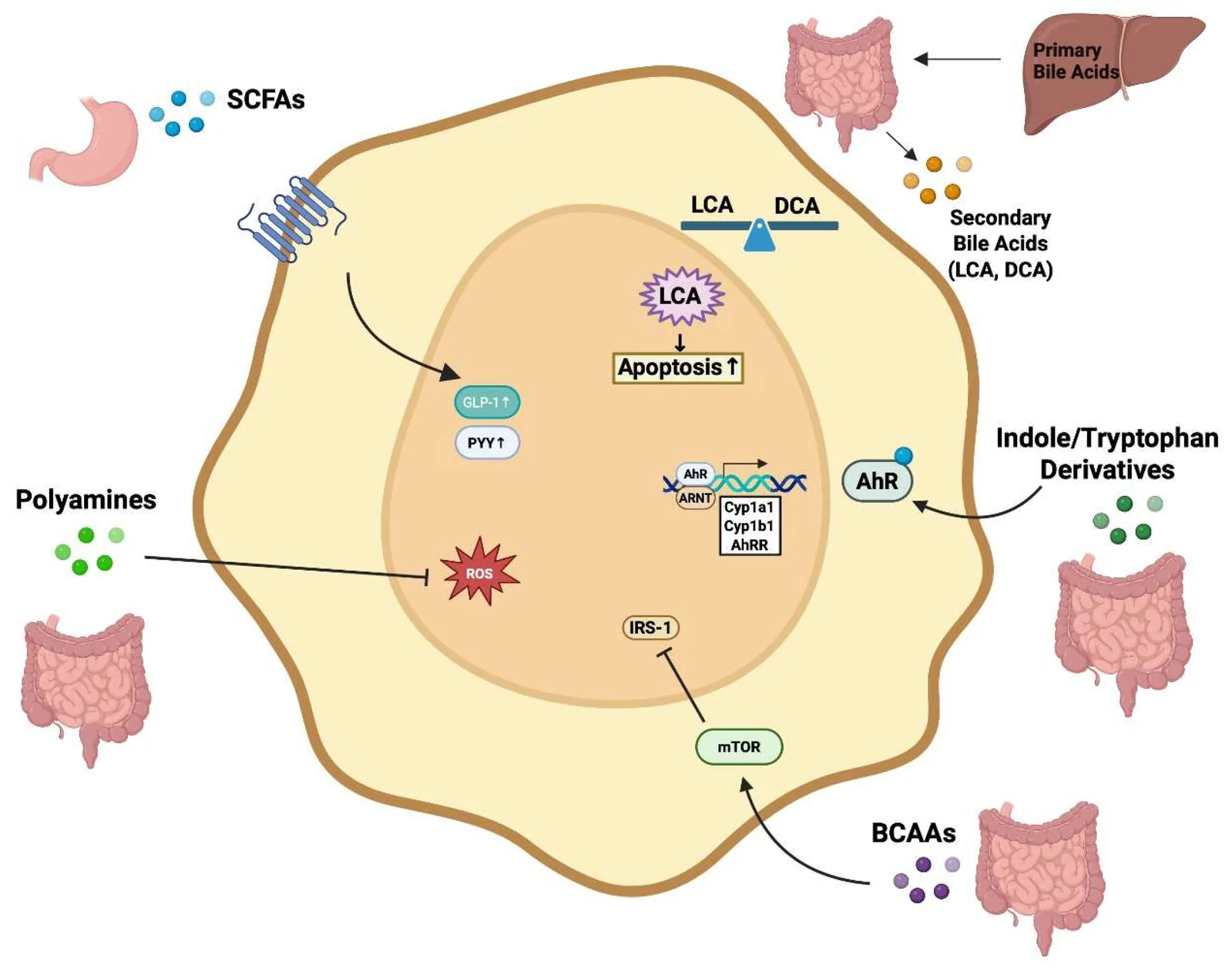
Major gut microbiota-derived metabolites and their effects on host cellular signaling pathways. Short-chain fatty acids (SCFAs), produced through microbial fermentation of dietary fibers, activate G-protein-coupled receptors (GPCRs) and stimulate the secretion of glucagon-like peptide-1 (GLP-1) and peptide YY (PYY). Gut microbes convert primary bile acids into secondary bile acids, including lithocholic acid (LCA) and deoxycholic acid (DCA), which influence intestinal homeostasis and cell survival. LCA may also induce apoptosis depending on the cellular context. Microbial indole and tryptophan-derived metabolites activate the aryl hydrocarbon receptor (AhR), which translocates to the nucleus, interacts with AhR nuclear translocator (ARNT), and regulates the transcription of target genes, including CYP1A1, CYP1B1, and AHRR. Polyamines may reduce intracellular reactive oxygen species (ROS), whereas elevated branched-chain amino acids (BCAAs) can activate mTOR signaling and impair insulin receptor substrate-1 (IRS-1) signaling, potentially contributing to insulin resistance.

Microbial metabolites are low-molecular-weight (<1500 Da) molecules that are derived from complex microbial metabolic pathways as intermediates or end products. These molecules play an important role in modulating everything from host immunity, microbial composition, and metabolic health to disease outcome [61,62,63]. They can be synthesized de novo by microorganisms (bacteria, viruses, fungi, actinomycetes, archaea, protozoa, etc.) or generated as spin-offs through the biotransformation of host dietary components. Several common microbial metabolites include short-chain fatty acids (SCFAs), bile acids, indole and tryptophan derivatives, polyamines, choline, branched-chain amino acids (BCAAs), etc. It is possible that the activity of certain microbial metabolites would change the chemotherapeutic efficiency of the therapy against cancer cells. Their effects on chemotherapy are not uniformly beneficial or detrimental, and the mechanism of action also varies (Figure 2, Table 2). For example, Urolithin A (UroA), which is generated by intestinal bacteria from ellagitannins and ellagic acid, is one such molecule. In a study performed by Ghosh et al., it was found that the combination of UroA and its synthetic analog UAS03 increased the effectiveness of 5-fluorouracil (5-FU) on 5-FU-resistant colorectal cancer cells by lowering the expression of several ABC transporters responsible for the export of drugs out of the cancer cell [64]. This resulted in an increase in 5-FU inside the cells and increased cytotoxicity. It was further noted that the mechanism behind this was related to the regulation of the FOXO3–FOXM1 pathway, and treatment with UroA/UAS03 and 5-FU inhibited the growth of 5-FU-resistant colorectal cancer. Such evidence suggests that some metabolites would be capable of changing the sensitivity to drugs by altering the drug transporter and related signaling pathways. Other metabolites, including secondary bile acids, short-chain fatty acids, indole derivatives, and polyamines, could have an impact on metabolism, apoptosis, immune response, and inflammation. SCFAs may inhibit HDACs, modify histone acetylation, apoptosis, immune cell differentiation, and transporter expression. Butyrate may sensitize some types of tumors to treatment at millimolar levels, but lower doses or targeted delivery can preserve barrier functions or increase expression of transporters. Tumor genotype and metabolism also impact the effect of butyrate. Valerate was proposed as another HDAC inhibitor among SCFAs, although a link between it and chemoresistance in patients has not been demonstrated yet [65]. Secondary bile acids act through FXR, PXR, and other receptors and may either strengthen barrier functions or cause DNA damage and cancer-associated signaling. Tryptophan metabolites act via AhR-mediated epithelial and immune pathways, while polyamines and bacterial exopolysaccharides impact proliferation, inflammation, and permeability. Thus, information about dosage, time of exposure, microbiome species, tumor localization, and host immune status should be provided for classification of metabolites as protective or resistance-enhancing compounds.

## 6. Molecular Mechanisms of Microbiota-Mediated Chemoresistance

**Figure 3:**
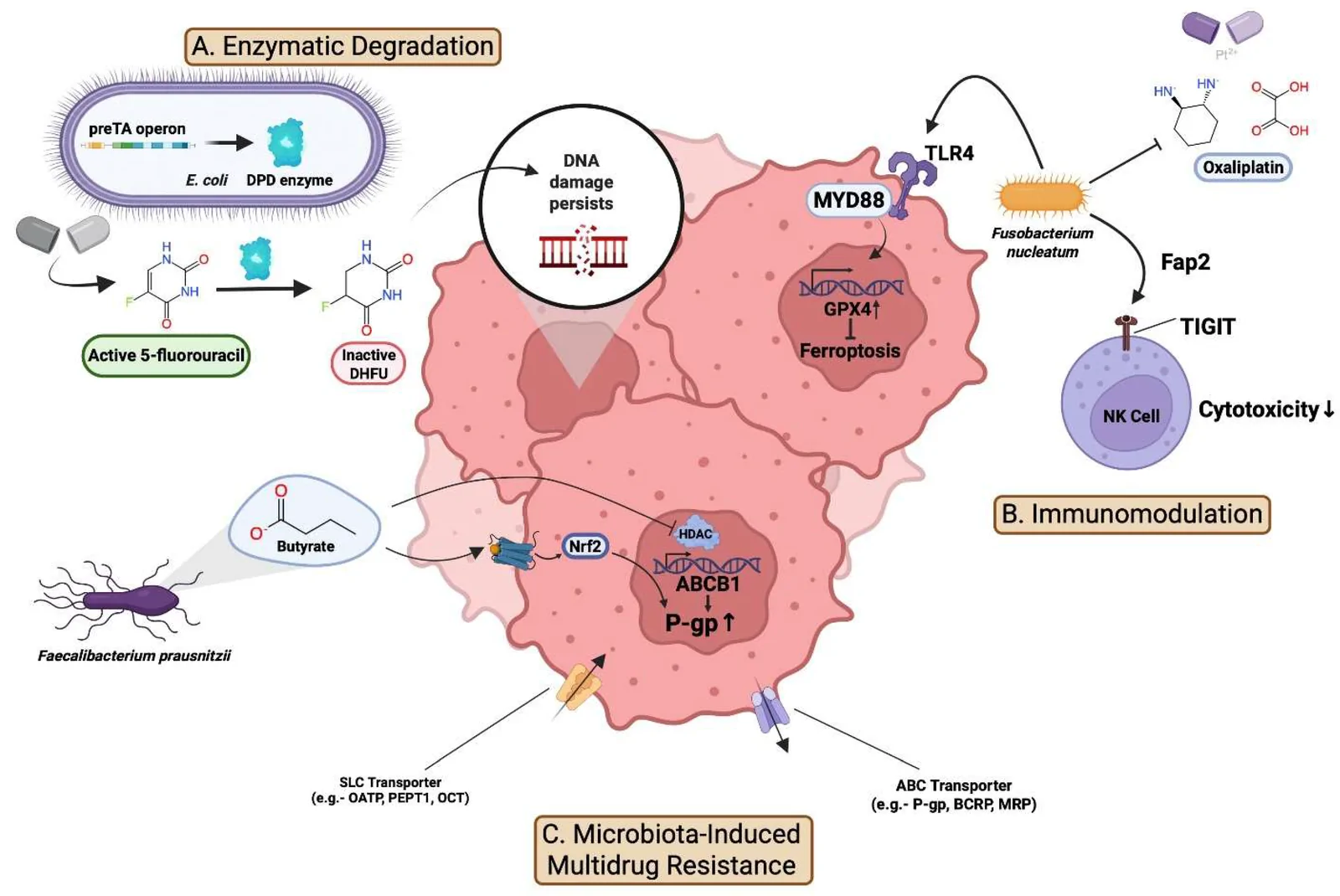
Molecular mechanisms of gut microbiota-mediated chemoresistance. (**A**) Enzymatic degradation: The bacterial preTA operon encodes dihydropyrimidine dehydrogenase (DPD), which converts active 5-fluorouracil (5-FU) into inactive dihydrofluorouracil (DHFU), reducing its therapeutic efficacy. (**B**) Immunomodulation: Fusobacterium nucleatum may promote oxaliplatin resistance by activating TLR4/MyD88 signaling, increasing GPX4 expression, and suppressing ferroptosis. It can also inhibit natural killer (NK) cell cytotoxicity through the interaction of bacterial Fap2 with the inhibitory receptor TIGIT. (**C**) Microbiota-induced multidrug resistance: Butyrate produced by Faecalibacterium prausnitzii may increase ABCB1 expression through HDAC inhibition and Nrf2-associated signaling, leading to increased P-glycoprotein (P-gp) expression and drug efflux. Solute carrier (SLC) transporters regulate drug uptake, whereas ATP-binding cassette (ABC) transporters mediate drug efflux, collectively influencing intracellular drug accumulation and chemotherapy response.

The chemoresistance caused by microbiota activity can be divided into different non-overlapping mechanisms (Figure 3). Firstly, enzymatic activity of microorganisms either activates or inactivates chemotherapeutic drugs. *Escherichia coli* makes 5-FU inactive by converting it into dihydrofluorouracil (DHFU) via a bacterial preTA operon-encoded enzyme. This reduces attenuation of DNA/RNA damage, thereby contributing to chemoresistance of tumor cells [68]. Gemcitabine is inactivated by members of intratumoral Gammaproteobacteria, which synthesize the enzyme cytidine deaminase (CDD) that converts gemcitabine into inactive 2′,2′-difluorodeoxyuridine [29]. An important indirect mechanism by which the microbiota affects drug response is through modulation of the host immune system. The signaling from microbes impacts the immune system through TLR-MYD88-NF-κB, STAT3, and inhibitory receptor signaling; evidence suggests that *Fusobacterium nucleatum* promotes resistance to oxaliplatin via activation of the TLR4/MYD88 signaling cascade. This pathway enhances autophagic activity and elevates GPX4 expression, resulting in inhibition of ferroptosis and improved survival of cancer cells exposed to chemotherapy [36]. The Fap2 protein of *F. nucleatum* binds to the human inhibitory receptor TIGIT to protect tumor cells from invasion of immune cells, thereby inhibiting NK cell cytotoxicity [69]. Moreover, microbial signaling supports the function of CSC and EMT programs. Finally, metabolic products influence chromatin structure, apoptosis, redox status, and transporters via HDAC, AhR, FXR/PXR, Nrf2, and FOXO3-FOXM1 signaling pathways. Gut microbiota and their metabolites have the ability to regulate the expression of two major families of transport proteins, namely, solute carrier (SLC) influx transporters and ATP-binding cassette (ABC) efflux transporters, which affect oral drug absorption and disposition [70]. Transporters including efflux transporters, P-glycoprotein (P-gp), breast cancer resistance protein (BCRP), and multidrug resistance-associated proteins (MRPs), as well as influx transporters, including organic anion-transporting polypeptides (OATPs), PEPT1, and OCTs, are regulated by gut microbiota and their metabolites. Butyrate produced by gut bacteria of the Clostridia group (*Faecalibacterium, Roseburia*) is known to inhibit histone deacetylases (HDACs). It leads to increased acetylation of histones and transcription of the ABCB1 gene responsible for the expression of the efflux transporter P-gp [66]. Additionally, butyrate activates several G-protein coupled receptors, including FFAR2/GPR43, FFAR3/GPR41, and GPR109A, leading to activation of Nrf2 through intracellular signaling pathways and upregulation of P-gp and MRP2. Metabolites of *Lactobacillus acidophilus* promote activation of the MAPK pathway with subsequent activation of AP-1 (c-Fos/c-Jun) transcription factors, stimulating the expression of P-gp [71]. Secondary bile acids (lithocholic acid (LCA), deoxycholic acid (DCA)), generated during the 7α-dehydroxylation process performed by microorganisms, serve as ligands for PXR and FXR [67]. PXR activation promotes expression of P-gp, BCRP, and MRP2, while FXR activation decreases NF-κB. Opposing actions of transporters do not necessarily contradict each other: for example, Urolithin A decreased the levels of certain ABC transporters in CRC models resistant to 5-FU [64] while butyrate or the signals from *Lactobacillus* increased the expression of ABCB1/P-gp or MRP2 [72].

## 7. Cancer-Specific Evidence of Microbiome-Associated Chemoresistance

Gut microbiota-associated chemoresistance has been documented in various studies across the globe. It has been found that intestinal microorganisms have been linked to various types of cancers. The evidence ranges from mechanistic preclinical studies to small clinical association cohorts.

### 7.1. Colorectal Cancer

5-fluorouracil has been one of the most widely prescribed drugs for the treatment of patients with CRC. 5-FU, a pyrimidine analog, is used to induce apoptosis in neoplastic cells [73]. Despite having promising efficacy against colorectal cancer, it has been found that nearly half of CRC patients display resistance to 5-FU due to the influence of harmful bacteria like *Fusobacterium nucleatum*, which activates the TLR4/MYD88 signaling pathway that results in upregulation of autophagy, tumor progression, cell survival, and suppression of immune cells [35]. Sometimes antibiotic treatment may induce gut dysbiosis, which elevates the population of *Staphylococcus* and *Clostridium,* thereby decreasing the abundance of *Bacteroides* and *Lactobacillus*. This results in chemoresistance to the platinum-based chemotherapeutic agent oxaliplatin [74]. A study conducted by Viaud et al. demonstrated that the efficacy of cyclophosphamide depends on the abundance of beneficial microorganisms [23].

### 7.2. Pancreatic Cancer

Gemcitabine is commonly employed for the treatment of pancreatic ductal adenocarcinoma (PDAC). However, the presence of pathogenic microorganisms such as Gammaproteobacteria (like *E. coli*) will change gemcitabine to its inactive form (2′,2′-difluorodeoxycytidine to 2′,2′-difluorodeoxyuridine) by virtue of the cytidine deaminase (CDA) enzyme [29]. FOLFIRINOX is a drug combination (5-fluorouracil, leucovorin, irinotecan, and oxaliplatin) commonly employed in the treatment of pancreatic cancer [75]. Use of FOLFIRINOX will affect the balance of intestinal microflora, with an increase in opportunistic microorganisms like *E. coli* and *Enterococcus* spp.

### 7.3. Breast Cancer

Breast cancer therapies such as trastuzumab reshape gut microbial communities, altering the relative abundance of *Clostridiales*, *Bacteroidales*, and *Firmicutes* [76]. A specialized subset of intestinal microorganisms can metabolize estrogens, termed the ‘estrobolome’. Dysbiosis causes alteration in estrogen metabolism, which may influence postmenopausal estrogen receptor–positive (ER+) breast cancer risk. Several gut bacteria possess β-glucuronidase and β-glucosidase enzymes that render estrogen into its deconjugated form, which is the biologically active form of estrogen [77]. Thus, accumulation of bioactive estrogen may increase the risk of ER-positive breast cancer.

### 7.4. Gastric Cancer

The strongest risk factor for the development of gastric cancer is infection with *Helicobacter pylori*. Two well-studied virulence factors of *H. pylori* are VacA and CagA, which have carcinogenic potential [78,79]. *H. pylori* infection upregulates POU5F1 gene expression, which further activates the SPP1 protein that promotes chemoresistance. A study conducted by Song et al. demonstrated that *H. pylori*-infected GC cell lines AGS and MKN-74 showed higher viability, migration, and invasion under DDP (cisplatin)/GEM (gemcitabine) treatment. *H. pylori* was also inoculated into immunocompetent mice, and it was observed that larger tumors were formed with reduced apoptosis. Silencing POU5F1 or SPP1 reduced tumor growth and increased the population of CD8^+^/CD3^+^ T cells in the xenograft tumors. *H. pylori* infection can also induce the expression of a specific miRNA, miR-135b-5p, which promotes DDP (cisplatin) resistance. The target of miR-135b-5p is Klf4, a tumor suppressor gene. Shao et al. showed that Hp-infected GC cell lines (MKN45, SNU1, and SNU601) elevated the expression of miR-135b-5p and displayed less sensitivity to cisplatin. They also conducted a similar experiment on Tff1-knockout mice, and the outcome was similar, where miR-135b-5p level was elevated and resistance to cisplatin was observed [37].

### 7.5. Lung Cancer

Despite the fact that there is no knowledge of the molecular mechanism underlying chemoresistance, a number of studies indicate a possible connection between gut microbiota and responsiveness and toxicity to chemotherapy in lung cancer [80,81,82]. Zhao et al. conducted research where a group of 64 patients with advanced or locally advanced lung cancer demonstrated higher baseline levels of *Acidobacteria*, *Granulicella*, *Streptococcus mutans*, and *Enterococcus casseliflavus* in those who were responders to platinum-based chemotherapy, whereas the group of non-responders contained high levels of such taxa as *Leuconostoc lactis*, *Eubacterium siraeum*, and *Rothia dentocariosa*. In addition, Zhang et al. noted that among 45 patients with non-small cell lung cancer (NSCLC) treated with platinum-based doublet chemotherapy (either carboplatin or cisplatin with gemcitabine or pemetrexed), the group of responders had higher enrichment of Bacteroides species, whereas gastrointestinal toxicity correlated with high levels of *Leuconostocaceae*. Another study of 60 patients with lung cancer suggested that baseline presence of genera such as *Prevotella*, *Megamonas*, *Streptococcus*, *Faecalibacterium*, and *Klebsiella* resulted in high risks of platinum-based gastrointestinal toxicity, while higher levels of *Bacteroides*, *Akkermansia*, *Bifidobacterium*, *Ruminococcus*, *Blautia*, and other genera were associated with low risks of such adverse events.

### 7.6. Melanoma

Evidence connecting the microbiome to conventional chemotherapy in melanoma is predominantly preclinical. However, the impact of the gut microbiota on chemoresistance development in the case of melanoma was poorly studied so far, although there is accumulating data that the microbiome may also regulate the efficiency of conventional chemotherapy [23,24,83,84]. The preclinical research proved that the gut microbiota is necessary for the maximal antitumor effect of cyclophosphamide. In mice bearing B16 melanoma, cyclophosphamide increased the permeability of the intestinal mucosa, causing the translocation of selected Gram-positive bacteria such as *Enterococcus hirae* to the secondary lymphoid organs, followed by Th1/Th17 and CD8^+^ T cell immune response and, consequently, improvement of the therapeutic effect of chemotherapy. At the same time, the antibiotic-induced dysbiosis inhibited the immune response and significantly decreased the antitumor activity of cyclophosphamide. In further studies, *Enterococcus hirae* and *Barnesiella intestinihominis* restored part of the cyclophosphamide response in antibiotic-treated mice. These commensals enhanced antitumor immune response through increasing the CD8^+^ T-cell infiltration into the tumor tissue, the T-cell response with production of IFN-γ, and the CD8^+^/Treg ratio, thus sensitizing the tumors to chemotherapy. Generally, dysbiosis is associated with dendritic cell hyporesponsiveness, reduction in antigen presentation, ROS generation by myeloid cells, and an immunosuppressive TME. Clinical studies of microbiota and anti-PD-1 therapy address a different treatment class and are discussed only as supporting evidence that microbiota can shape systemic antitumor immunity.

### 7.7. Hematological Malignancies

Chemotherapy-induced gut microbiota dysbiosis is being linked as a cause of chemotherapy resistance in acute myeloid leukemia (AML) [85]. Antibiotics and chemotherapy result in reduced microbiome diversity, a decrease in the population of SCFA-producing bacteria such as *Faecalibacterium*, and an increase in pathogenic bacteria such as Enterococcus and Fusobacterium, leading to reduced levels of IL-17/IL-22 cytokines, causing disruption of mucosal immunity. In addition to this, some bacteria present in the gut have similar reductases that deactivate anthracyclines like daunorubicin.

## 8. Impact of Antibiotics and Dysbiosis on Chemotherapy Outcomes

Antibiotic-related dysbiosis is also associated with cancer progression in different types of cancer. Antibiotics can reduce microbial diversity, eliminate beneficial functions, select resistant organisms, and modify chemotherapy-associated toxicity or efficacy. The effect depends on antimicrobial spectrum, timing, indication, baseline microbiota, and cancer treatment. The gut microbiome plays a pivotal role in drug metabolism; however, its influence on drug metabolism remains poorly understood. Gut microbiota and microbial metabolites directly influence anti-tumor response and clinical effectiveness of ICI therapy and its adverse effects [86]. However, the detailed molecular mechanisms involved in gut-microbiota-mediated immunomodulation are yet to be elucidated. Probably, the microbial antigens non-specifically activate peripheral immune cells to enhance T-cell cytotoxicity to support tumor-specific responses. However, Observational studies linking antibiotic exposure to poorer outcomes are susceptible to confounding by infection severity and clinical status; they should not be interpreted as proof that antibiotics directly cause chemoresistance. Antimicrobial spectrum, timing, indication, baseline microbiota, and cancer treatment. Experimental models nevertheless support several plausible routes, including impaired myeloid-cell ROS production, reduced commensal-driven T-cell priming, altered SCFA production, and expansion of pathobionts. Antibiotic stewardship is therefore important, but clinically indicated treatment should not be withheld on the basis of microbiome associations. Previous work established both the influence of non-antibiotic drugs on gut microbiota composition and variation in gut microbiome composition, altering drug availability and efficacy in different disease conditions, including cancer. The first study on a murine system sheds light on the fact that anti-CTLA-4 treatment efficacy was dampened in germ-free mice. A similar result was also observed in specific germ-free mice that received antibiotic treatment [87]. However, after oral inoculation of *B. fragilis* along with *Burkholderia cepacia* or *Bacteroides thetaiotaomicron,* resulting betterment of dendritic cell (DC) maturation within the tumor, followed by a heightened Th1 response. Thus, a change in microbiota improved the anti-tumor function of anti-CTLA-4 therapy. Another report suggested that higher abundance of *Bifidobacterium* species was associated with enhanced PD-L1 blockade therapy in a murine system [45]. Finally, Routy et al. revealed that patients treated with PD-L1 or PD-1 blockade therapy who received antibiotics before, during, or after ICI therapy exhibited reduced progression-free survival compared with the antibiotics-untreated group. Subsequent research demonstrated a positive correlation between the presence of a few gut microbial taxa (i.e., *Bifidobacterium longum*, *Enterococcus faecium*, and *B. thetaiotaomicron*) and ICI therapy, while *E. coli* had an opposite correlation [46]. These observations do not establish the same mechanisms for chemotherapy.

Clinical chemotherapy studies also suggest that antibiotic-associated dysbiosis may influence outcomes, although causality remains uncertain. Dysbiosis is accompanied by several health issues like inflammatory bowel disease (IBD), obesity, Gastroesophageal Reflux Disease (GERD), and irritable bowel syndrome (IBS). It is also associated with several mental illnesses (i.e., anxiety and depression) and metabolic diseases like non-alcoholic fatty liver disease (NAFLD), type 2 diabetes, and cancer. A report by Corona et al. revealed that multiple myeloma (MM) patients with exposure to any broad-spectrum, non-prophylactic antibiotic within 30 days when treated with CD3/B-cell maturation bispecific antibodies (BaAb, n = 155) showed significantly shorter OS and PFS BUT higher relapse. Deeper analysis demonstrated a noticeable reduction in the *Roseburia* and *Eubacterium* genera, resulting in lower serum short-chain fatty acid (SCFA) concentrations. Hence, this study showed that antibiotic-induced dysbiosis before BsAb therapy is linked with diminished immunity and inferior therapeutic outcomes for MM patients. Maximum antibiotics prescribed for bacterial infections can activate the SOS response [88]. For example, quinolones (i.e., ciprofloxacin) hinder bacterial topoisomerases II and IV, resulting in double-stranded DNA breaks that trigger the SOS response, suggesting that combining antimicrobial prophylaxis therapy with chemotherapy may inadvertently increase the risk of de novo antibiotic resistance in gut microbiota. This reverses the future mitigation of bacterial infections, causing higher mortality rates in CRC patients due to antibiotic failure and remaining undetected [89]. This matches recent studies revealing that cancer patients undergoing chemotherapy, along with antimicrobial prophylaxis, exhibit poorer overall survival (OS) than those without prophylaxis [90]. For example, Chambers et al. al demonstrated reduced OS in epithelial ovarian cancer patients who underwent antibiotic therapy during platinum chemotherapy. Specifically, antibiotics killing Gram-positive bacteria significantly deteriorated patients’ OS (35.0 vs. 62.4 months) [91]. Another study on non-small-cell lung cancer (NSCLC) patients documented that chemotherapy followed by one month of antibiotic treatment significantly reduced OS compared to those who did not receive antibiotics (13.8 vs. 17.6 months, *p* < 0.001) [92]. Hence, antibiotic treatment prior to chemotherapy is steadily linked with poor OS for cancer patients. Sheflin et al. earlier documented that imbalance in the gut microbiome causing chronic infections endorses tumorigenesis via persistent inflammation, NF-κB activation, activation via Toll-like receptor signaling, and generation of genotoxic metabolites, finally leading to immune evasion [93]. Using high-throughput sequencing and multi-omics methods, precise dysbiotic signatures are identified and characterized across various cancer types, focusing on the enrichment of the *Fusobacterium nucleatum* population in colorectal cancer (CRC) and the recognized involvement of *Helicobacter pylori* in gastric carcinogenesis [94]. Thus, the gut microbiome exhibits a dual role, i.e., as a cancer-associated dysbiosis marker as well as a dynamic regulator of host immunity, inflammation, and metabolism to monitor therapeutic responsiveness [95]. Future studies should prospectively document indication, class, dose, timing, and duration of antibiotic use and analyze chemotherapy separately from bispecific antibodies or immune checkpoint inhibitors.

## 9. Clinical Applications and Current Challenges

### 9.1. Microbiome as a Predictive Biomarker

The gut and intratumoral microbiomes are increasingly recognized as modulators of chemotherapeutic efficacy and resistance, and also, they are being evaluated as biomarkers of chemotherapy response. The ‘TIMER’ mechanism (Translocation, Immunomodulation, Metabolism, Enzymatic degradation, and Reduced diversity) has earlier been suggested as the potential mechanisms by which the gut microbiome influences the efficacy of chemotherapeutic agents [22]. Candidate predictors include taxonomic profiles, microbial genes and enzymes, metabolite concentrations, and functional readouts such as fecal β-glucuronidase activity. The molecular insights from profiling the microbiome via next-generation sequencing such as whole-genome shotgun and 16S rRNA sequencing, multi-omics data including genomics, transcriptomics, proteomics, metabolomics, and exposomics, and machine learning algorithms identified microbial signatures enabling the customization of therapy based on the distinct microbiome makeup of the individual [22,96]. Across multiple solid tumors, a plethora of evidence revealed that specific microbial taxa and their metabolites, microbiome modulation through antibiotics, probiotics, prebiotics, postbiotics, diet, or fecal microbiota transplantation, and microbiome-derived signals are either associated with chemosensitivity/chemoresistance or alter treatment responses in preclinical and clinical settings, suggesting their potential as non-invasive predictive biomarkers for treatment planning and outcome [22,23,26,29,35,84,96,97,98,99,100,101,102,103,104].

### 9.2. Limitations of Current Studies

In the age of precision medicine, assessing gut dysbiosis in patients and utilizing microbiota modulation strategies may represent a promising strategy for enhancing responses to anticancer treatments and improving treatment outcomes by overcoming chemoresistance. Despite promising progress, numerous obstacles impede the clinical implementation of microbiome-based therapies, such as differences in microbiome makeup, heterogeneous sequencing methodologies, absence of standardized methods, regulatory challenges, safety issues, and ethical concerns [105,106]. Sample collection and handling, storage, DNA extraction, sequencing platform, bioinformatic pipeline, contamination control, and compositional-data analysis can each change the reported microbiome signature. Low-biomass tumor samples are especially vulnerable to reagent and environmental contamination. 16S rRNA sequencing is cost-effective but provides limited strain and functional resolution; shotgun metagenomics improves gene-level inference but is more expensive and analytically demanding [107]. Meta-transcriptomics, metabolomics, and host immune profiling can improve mechanistic interpretation. Machine-learning models may integrate all layers, but overfitting, batch effects, missing external validation, and population-specific signatures remain major barriers. Studies should include method extraction blanks, standardized metadata, absolute microbial load measurements where possible, and independent validation. Among these challenges, inter-individual variation in microbial composition of the gut poses the major obstacle to developing universal microbiome-based biomarkers for effective therapy. Various elements such as host heredity, age, sex, diet, geographical region, race, nutritional status and cancer cachexia, bowel function, comorbidities, and co-medications such as proton-pump inhibitors, NSAIDs, laxatives, metformin, and antibiotics affect microbiome composition, resulting in discrepancies in research findings. This variability makes it challenging to identify consistent microbial signatures to tackle therapy resistance and predict successful treatment efficacy [106,108]. Moreover, Alpha diversity alone does not define eubiosis or dysbiosis; functional capacity, community composition, and spatial organization are also important. Most mechanistic findings derive from cell culture or animal models, whereas clinical cohorts are often small and heterogeneous.

### 9.3. Microbiome-Directed Interventions and Safety

Prebiotics, probiotics, and synbiotics are reported to support gut barrier integrity, SCFA production, and recovery from dysbiosis. However, due to the heterogeneous nature of oncology, they have failed to overcome chemoresistance [95]. Traditional medicines and plant-derived polyphenols work through microbial biotransformation, partly through their active constituents; dose, pharmacokinetics, and product quality require careful evaluation. While FMT and live biotherapeutic products allow for more microbial manipulation, they pose dangers related to pathogen transfer, bacteremia in immunocompromised hosts, antibiotic resistance gene transfer, unpredictable engraftment, and possible ecological consequences [109,110]. In a study by Davar et al., it was found that responder-derived FMT modulated the intestinal microbiome, increased the density of CD8^+^ cytotoxic T cells, and subsequently reduced immunosuppressive myeloid cells in patients who were previously resistant to pembrolizumab [109]. Yang et al. conducted a clinical study where they orally administered GB104 live biotherapeutic product (LBP) to nine colorectal cancer patients in a phase 1 trial, which was composed of a single bacterial strain of *Lactiplantibacillus plantarum.* Monotherapy of GB104 provided dose-dependent improvement in bowel function, while minimizing changes in microbial diversity [110]. Donor testing, strain-level characterization, manufacturing process control, pharmacovigilance, and clinical trial studies are also very critical to maintain safety.

### 9.4. Emerging Environmental and Precision-Oncology Considerations

The impact of climate change on food systems, infectious disease nature, antimicrobial agents, and environmental exposures may indirectly reshape human microbiomes [111,112]. Micro- and nanoplastics impacts on barrier functions and microbial populations are also being actively studied, but direct evidence that these exposures cause chemotherapy resistance is currently insufficient. Poor outcomes may be certain, particularly in lower- and middle-income countries, where late diagnoses, infectious comorbidities, environmental exposures, sequencing expense, and limited access to regulated microbiome products may worsen all implementation gaps. Artificial intelligence and machine learning have promise to aggregate microbiome data, metabolomics, tumor genomics, and clinical data, and CRISPR and synthetic biology may help target microbial function removal or modification, but these methodologies require external validation, transparent and accessible models, containment controls, and rigorous oversight before clinical use.

## 10. Conclusions

There is growing evidence to support the notion that the microbiome impacts chemotherapy resistance, antitumor immunity, pharmacotherapy effects, and pharmacotoxicity. The ways by which microbiota affect chemotherapeutic agents include biotransformation, immune modulation, damage to barriers, biofilm formation, signaling between CSC and EMT cells, and metabolite-mediated regulation of chromatin and transporter functions. Clear cases of causality have been observed in pre-clinical studies, mainly due to cytidine deaminase-dependent degradation of gemcitabine in bacteria and the mechanisms of resistance to chemotherapies associated with *Fusobacterium nucleatum* [29,35]. These clinical correlations between the two hold promise but are insufficient to exploit the microbiota in directing the process of chemotherapy. Future research should include prospective, properly powered cohorts; proper sampling; correlative multi-omics; validation of microbial enzymes and metabolites; and therapy-specific outcomes that can distinguish efficacy from toxicity. Microbiome medicine needs to advance from the manipulation of communities towards defined and mechanism-based products with proper safety assessment. FMT, probiotics, synbiotics, live biotherapeutics, phages, and engineered strains may eventually complement precision oncology, but benefits must be demonstrated for specific cancer-drug contexts and balanced against infection, gene-transfer, ecological, and regulatory risks. Together, these findings support a shift from simply designating the microbiome as a biomarker towards actively manipulating it as a therapeutic component of precision oncology, although clinical studies on a large scale are still required to establish standardized and patient-specific approaches.

## Figures and Tables

**Table 1:** Microbiota-mediated changes in chemotherapy efficacy or toxicity.

Sl. No.	Chemotherapeutic Agent	Gut Bacteria	Effect on Therapeutic Efficacy	Evidence Level	Reference
1	Gemcitabine	Gammaproteobacteria	isoform of bacterial cytidine deaminase converts active gemcitabine into the inactive metabolite 2′,2′-difluorodeoxyuridine, influencing chemoresistance.	Patient specimens; mouse and enzymatic studies	[29]
2	Gemcitabine	*Enterococcus faecium*	cytidine deaminase deaminates the drug into the inactive form, modulating gallbladder cancer	Structural/enzymatic; clinical relevance unconfirmed	[30]
3	Irinotecan	Bacterial β-glucuronidases (multiple gut taxa)	Reactivate SN-38 from SN-38G in the gut lumen, increasing local intestinal toxicity; this is not evidence of reduced systemic antitumor efficacy.	Animal mechanistic toxicity studies	[31,32]
4	Doxorubicin	*Raoultella planticola*	Deglycosylates doxorubicin into 7-deoxyrubicinol/7-deoxyrubicinolone, leading to less systemic distribution.	C. elegans/preclinical	[33]
5	5-fluoro-2′-deoxyuridine, 5-trifluorothymidine, 5-halogenated 2′-deoxyuridines	*Mycoplasma hyorhinis*	Bacterial thymidine phosphorylase converts the drugs to their inactive bases and reduces their cytostatic efficacy in breast cancer	Cell culture/preclinical	[34]
6	5-FU	*Fusobacterium nucleatum*	Promotes resistance through TLR4/MYD88 signaling, miRNA-dependent autophagy, and immune modulation.	CRC cells, mouse models, and patient association	[35]
7	Oxaliplatin	*Fusobacterium nucleatum*	Suppresses ferroptosis through E-cadherin/β-catenin/GPX4 signaling.	Cell and animal mechanistic studies	[36]
8	Cisplatin	*Helicobacter pylori*	Promotes resistance-associated miR-135b-5p/KLF4 and POU5F1-SPP1 signaling in gastric cancer models.	Cell lines and mouse models	[37]

**Table 2:** Microbial metabolites and pathways relevant to chemotherapy response.

Metabolite	Representative Source	Host Target/Pathway	Potential Effect	Evidence Context	Reference
Butyrate	*Faecalibacterium*, *Roseburia* and other fermenters	HDACs; GPR41/43; GPR109A; Nrf2	May alter apoptosis, Treg differentiation, and ABC transporters; direction depends on dose and model	Mainly cell and animal studies	[43,66]
Urolithin A	Ellagitannin-transforming gut bacteria	FOXO3-FOXM1; ABC transporters	Sensitized 5-FU-resistant CRC models	Cell culture and mouse tumor models	[64]
Secondary bile acids	7α-dehydroxylating bacteria	FXR, PXR, NF-κB; DNA-damage pathways	May regulate transporters and inflammation or promote tumor-supportive signaling	Preclinical; context-dependent	[67]
Tryptophan derivatives	Indole-producing bacteria	AhR and epithelial-immune pathways	May strengthen barrier immunity or alter tumor-associated immune signaling	Predominantly preclinical	[6,62]
Valerate and other SCFAs	Microbial fermentation	HDAC inhibition and chromatin regulation	Potential epigenetic modulation; direct chemoresistance evidence limited	Mechanistic/emerging	[65]

## Data Availability

No new data were created or analyzed in this study. Data sharing is not applicable to this article.
